# Silymarin constrains diacetyl-prompted oxidative stress and neuroinflammation in rats: involvements of Dyn/GDNF and MAPK signaling pathway

**DOI:** 10.1007/s10787-022-00961-9

**Published:** 2022-04-02

**Authors:** Manar Mohammed El Tabaa, Hamdi M. Aboalazm, Mohamed Shaalan, Naglaa Fathy Khedr

**Affiliations:** 1 Pharmacology & Environmental Toxicology, Environmental Studies & Research Institute (ESRI), University of Sadat City, Minofia Governorate, Sadat city, Egypt; 2 Biochemistry, Environmental Studies & Research Institute (ESRI), University of Sadat City, Sadat City, Egypt; 3 Pathology Department, Faculty of Veterinary Medicine, Cairo University, Giza, Egypt; 4 Biochemistry, Faculty of Pharmacy, Tanta University, Tanta, Egypt

**Keywords:** Silymarin, Diacetyl, Neuroinflammation, Dynorphin, GDNF, MAPK pathway

## Abstract

Neuroinflammation, a major component of many CNS disorders, has been suggested to be associated with diacetyl (DA) exposure. DA is commonly used as a food flavoring additive and condiment. Lately, silymarin (Sily) has shown protective and therapeutic effects on neuronal inflammation. The study aimed to explore the role of Sily in protecting and/or treating DA-induced neuroinflammation. Neuroinflammation was induced in rats by administering DA (25 mg/kg) orally. Results revealed that Sily (50 mg/kg) obviously maintained cognitive and behavioral functions, alleviated brain antioxidant status, and inhibited microglial activation. Sily enhanced IL-10, GDNF and Dyn levels, reduced IFN-γ, TNFα, and IL-1β levels, and down-regulated the MAPK pathway. Immunohistochemical investigation of EGFR and GFAP declared that Sily could conserve neurons from inflammatory damage. However, with continuing DA exposure during Sily treatment, oxidative stress and neuroinflammation were less mitigated. These findings point to a novel mechanism involving the Dyn/GDNF and MAPK pathway through which Sily might prevent and treat DA-induced neuroinflammation.

**Supplementary Information:**

The online version contains supplementary material available at 10.1007/s10787-022-00961-9.

## Introduction

One of the central nervous system (CNS) problems that has universally gained much attention in the recent years is neuroinflammation (Lassmann 2021). Neuroinflammation has been found to be a key hallmark associated with different CNS disorders such as Alzheimer's disease, Parkinson's disease, schizophrenia, and depression (Skaper et al. 2018; Stephenson et al. 2018). It is an inflammatory response which may exacerbate tissue damage, nerve cell death, and potentially affect the structure and function of brain or spinal cord (DiSabato et al. 2016).

When neurons become inflamed as a result of brain injuries or the passage of potentially hazardous substances or pathogens across the blood–brain barrier (BBB) (Milatovic et al. 2011), CNS resident cells (microglia and astrocytes) become activated. This results in the generation of more toxic reactive free radicals (ROS) as well as a variety of inflammatory modulators such as tumor necrosis factor alpha (TNFα), and interleukin-1 beta (IL-1β) which may compromise BBB function (da Fonseca et al. 2014).

Both ROS and inflammatory cytokines ensure the continuity of inflammatory cascade via activating the mitogen-activated protein (MAP) kinases signaling pathway (Hayden and Ghosh 2014). MAP kinases are made up of three active kinases: an MAPK, an MAPK kinase (MAPKK), and an MAPKK kinase (MAPKKK) (Cargnello and Roux 2011). For an MAPK, there are three major subfamilies, namely extracellular signal-regulated kinase (ERK), c-Jun-amino-terminal kinase (c-JNK), and p38 MAP kinase (p38-MAPK) that have been detected to play crucial regulatory functions during inflammation (Manzoor and Koh 2012). ERK 1/2 is responsible for cell growth, differentiation, and meiosis and regulated by the Raf family of MAPKKK and MEK1/2 of MAPKK, while JNK and p38 MAPK are responsible for inflammation, apoptosis, cell differentiation, and cell cycle regulation and regulated by MKK 4/7 and MKK 3/6, respectively (Roux and Blenis 2004).

In addition to the proinflammatory effect, CNS microglia after being activated can also exert a neuroprotective role through secreting various neurotrophic factors (Suzumura 2017), such as glial cell line-derived neurotrophic factor (GDNF), which can affect the inflammatory microglial functions (Pöyhönen et al. 2019). GDNF may efficiently reduce the release of inflammatory mediators and ROS being generated as a result of microglial activation (Lima Giacobbo et al., 2019; Rocha et al. 2012). Moreover, new insight reports that endogenous dynorphin (Dyn) can also possess potent anti-inflammatory properties through turning the microglial effect from being pro-inflammatory to anti-inflammatory one (Liu et al. 2020).

During the current century, researchers pointed out that exposure to diacetyl (2, 3-butanedione; DA) might induce neurotoxicity because of its capability to easily cross the BBB (Das and Smid 2019; More et al. 2012). DA is a food flavoring agent that is widely present in human food products including dairy products, roasted coffee and caramel. It is often added as a condiment to microwave buttered popcorn to improve the food smell and taste by providing a butter-like flavor (Clark and Winter 2015).

Although DA is regarded by United States Food and Drug Administration (FDA) as ‘Generally Recognized As Safe’ (GRAS), many investigations have shown that DA intake may cause uncontrolled acetylation, altering the protein structure and, subsequently, inducing a state of oxidative stress which may promote a series of lung, kidney, and liver tissue damage (Brass and Palmer 2017; Thimraj et al. 2019). However, for CNS, there is not enough evidence about the mechanisms by which DA may harm the nervous tissue.

Simultaneously, there has been enthusiasm toward the efficacy of flavonoids in maintaining the neuronal function within the brain (Ayaz et al. 2019). Flavonoids possess a pluralism of neuroprotective actions varying from enhancing cognitive and behavioral brain function, protecting neurons against either age or neurotoxins, to suppressing neuroinflammation (Singh 2019). One of the remarkable plant-derived flavonoids considered as an eminent worldwide hepatoprotective drug is silymarin (Sily) (Freitag et al. 2015; Mengesha et al. 2021). Surprisingly, Sily has also been reported as a potent neuroprotective agent via enhancing neurons function and stimulating their regeneration (Raza et al. 2011; Mehri et al. 2016). Sily, as a flavonoid, has the potential to reduce oxidative stress and the production of inflammatory cytokines in the brain (Lovelace et al. 2015; Surai 2015).

Overall, this study will discuss the potential protective and therapeutic efficacy of Sily against the oxidative–inflammatory cascade and associated damage induced following DA intake in the brain.

## Materials and methods

All the experimental proceedings were approved by the research ethical Committee of Faculty of Pharmacy-Tanta University, Egypt. The animal experimentation was conducted in compliance with the guidelines of Institutional Animal Care and Use Committee.

### Animals

Fifty-six adult male *Wistar* rats weighing 120–130 g were supplied by the Experimental Animals Production unit of VACSERA (Giza, Egypt). The rats were housed in ventilated free pathogen plastic cages at room temperature with 40–60% humidity during the 12 h light/dark phase. All animals were retained free with a standard laboratory diet and filtered water ad libitum.

### Chemicals and drug

Diacetyl (2,3-butanedione, DA) (97%, 431–03–8) was purchased from Sigma-Aldrich Co. Inc. (St. Louis, MO, USA). Silymarin (Sily) (Hepaticum® oral suspension, a branded medicine made of micronized silymarin) was purchased from Medical Union Pharmaceuticals (MUP), Ismailia, Egypt. Based on drug label, each 1 ml contains 10 mg of dry milk thistle (*Silybum marianum*) extract standardized to silybin (A + B) 60.33%, isoslybin (A + B) 11.14% and silydianine and silychrisine 28.34%, free from sugar and glycol contents. Colorimetric assay kits for determining the levels of malondialdehyde (MDA) (MD2528) and nitric oxide (NO) (Cat No: NO2533), as well as total antioxidant capicity (TAC) (TA2513) were obtained from Bio-Diagnostics Co. (Dokki, Giza, Egypt). Rat ELISA kits for interlukin-10 (IL-10) (SEA056Ra), glial cell line derived neurotrophic factor (GDNF) (SEA043Ra), big dynorphin (Dyn) (CEB187Ra), interferon gamma (IFN-γ) (SEA049Ra), tumor necrosis factor alpha (TNFα) (SEA133Ra), and interlukin-1β (IL-1β) (SEA563Ra) were obtained from Cloud-Clone Corp Co. (Houston, USA). Colorimetric assay kit for measuring acetylcholinesterase activity (AChE; #K764) was obtained from BioVision Inc. (Milpitas, USA). Total protein extraction kit (NBP2-37,853) was obtained from Novus Biologicals, LLC, USA. Pierce™ bicinchoninic acid (BCA) protein assay kit was obtained from Thermo Fisher Scientific Inc., USA. Rabbit monoclonal anti-p44/42 MAPK (Erk1/2) (# 4695), anti-phospho-p44/42 MAPK (ERK1/2) (pThr202/Tyr204; #4377S), anti-JNK (#9252), anti-phospho-JNK (pThr183/pTyr185; #4671S), anti-p38MAPK (##8690), anti-phospho-p38MAPK (Thr180/Tyr182; #9215S), anti-β-actin (#4970S) and anti-rabbit IgG HRP-linked antibody (#7074S) were gained from Cell Signaling Technology, Inc., USA. Moreover, rabbit polyclonal anti-nuclear factor-κB (NF-κB) (ab231481), anti-glial fibrillary acidic protein (GFAP) (ab68428), and anti-epidermal growth factor receptor (EGFR) (ab40815) antibodies were purchased from Abcam, USA. Other chemicals of analytical grade were available commercially, or as prescribed.

### Induction of neuroinflammation

Neuroinflammation was induced in rats via administering25 mg diacetyl (DA)/kg/day orally for 15 days (Bawazir 2016), dissolved in distilled water (D.W.) in a ratio of 1:5 v/v (Morgan et al. 2008). Since the concentration of DA was 981 mg/mL, each rat received 0.15 mL of 0.03 mL DA + 0.12 DW/kg/day.

### Design of the experimental protocol

**Scheme 2.1:**
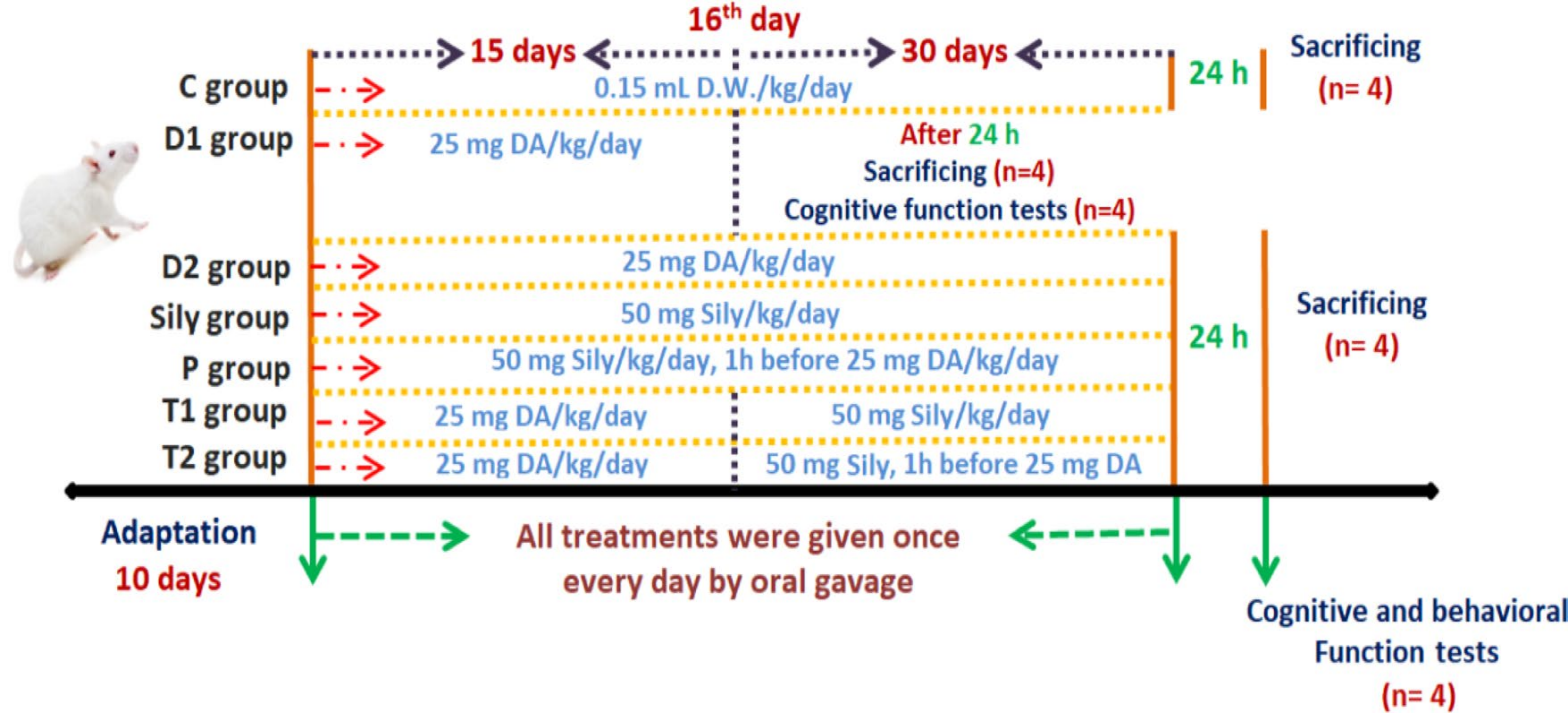
Experimental design

After 10 days of experimental acclimatization, rats were randomly divided into seven main groups (*n* = 8) as follows as in Scheme 2.1. Control (C) group: normal rats received only 0.15 mL D.W./kg/day as vehicle. Diacetyl-15 (DA1) and − 30 (DA2) groups: rats received 25 mg DA/kg/day for 15 and 30 days, respectively. Silymarin (Sily) group: rats received 50 mg Sily/kg/day for 15 days (Yardım et al. 2021). Protected (P) group: rats received with 50 mg Sily/kg/day, 1 h before administering 25 mg DA/kg/day for 15 days (Sornsuvit et al. 2018). Treated-15 (T1) group: rats were monotreated 25 mg DA/kg/day for 15 days, and on day 16 DA was stopped and replaced by 50 mg Sily/kg/day for another 15 days. Treated-30 (T2) group: rats were monotreated with 25 mg DA/kg/day for 30 days, but on day 16 rats received 50 mg Sily/kg/day for the next 15 days along with DA. All treatments were given once every day via oral gavage (P.O.). Animals from each group were weighed using a digital balance at the start of the experiment and at the end of each week (i.e., at the 1st, 2nd, 3rf and 4th week). Following 24 h from the end of the experiment, four rats from each group were randomly selected for evaluating the cognitive and behavioral functions.

### Cognitive and behavioral function test

#### Morris water navigation task

Morris water task was performed to test the effect of brain lesions in the cortical regions (responsible for recording the information concerning animal environment and orientation) on spatial learning and memory (Morris 1984). The task was carried out based on the use of a praxic strategy for getting out of the maze via assessing the ability of each rat in remembering the movements needed to find the correct path for the invisible submerged platform, onto which the rat could climb and escape. Rats were placed in a large rectangular glass tank (40 cm × 70 cm in diameter × 60 cm in height) with specific four corners, which could help them to determine the dimensions and orientations easily without using any cue. The tank was first divided into four equal quadrants (A), (B), (C) and (D) prior to filling it with warm water, where each quadrant had only one specific corner. The platform was submerged under the water at 2 cm depth and fixed in only quadrant (A) during all trials. Firstly, each rat was trained once a day for four consecutive days to locate the hidden platform in the clear water during 60 s. If the rat was confused and could not remember the location, it should be gently oriented and allowed to find it for an additional 30 s. During the test trial, milk powder was initially added to make the water opaque and to block the vision of the targeted platform. Then, each animal was tested three times a day to locate the hidden platform depending on the spatial orientation to the tank corners within 60 s. Each time, animals were placed into the cloudy water facing the tank wall in one of the three quadrants (B), (C) and (D) that did not contain the platform. The cognitive performance of rats during the swimming was then assessed by measuring the escape latency time (s), which was required for a rat to detect the hidden targeted platform. For each rat, the time of three test trials per a day was recorded and their average calculated.

#### Y-maze test

Y-maze test was commonly used for assessing the recognition memory of rodents by measuring the time spent in exploring the area with a food reward (Maurice et al. 1994). Testing was performed in a Y-shaped maze made of three identical glass arms (1), (2), and (3) with about 130° angle between them. Each arm was 45 cm × 30 cm in diameter and 60 cm in height and blocked off from the end to prevent rats from escaping out of the maze. First, each rat was familiarized with the maze before introducing the food. Then, two identical plastic plates were placed at arms (1) and (2) only. The plate of arm (1) was left empty, while that of arm (2) was filled with Rumi cheese as a food reward for rats. Each rat was trained for 3 min once a day for three consecutive days to detect the plate with food reward and distinguish it from the empty one. During the test session, animals were placed at the entrance of an empty arm (3) and allowed to move freely for 3 min to explore the other two arms and to find the way for a food reward. If the rat directly chose to go toward the arm in which a plate with a food reward existed, it chose “correctly”. But, if it chose the arm with an empty plate, it chose “incorrectly”. The percentage (%) of correct arm choice was measured by dividing the maximum number of entries into the arm with food reward by the total number of entries into all three arms X 100. Also, exploring time % was recorded by dividing the time spent by each rat for exploring the arm with food reward by the total time spent in all three arms multiplied by 100.

#### Climbing pole test

A pole test was used to investigate the rodent motor coordination. A stainless steel rod (60 cm length × 1 cm diameter) of a support laboratory holder for burettes was used and located inside a glass house. The rod was equally divided by a white skin plaster into upper and lower halves (each of 30 cm length). Rats were placed with upward-directed heads on the top of the rod, to which they can grasp through their four paws. The animals were trained for 60 s to climb down the rod, three consecutive times with a delay interval of 30 s in between. Lastly, each rat was tested for 30 s to climb down with head directed downward. The percentage (%) of rats able to turn their head 180^◦^ to the total number of rats per each group was evaluated. In addition, the latency to climb down the rod into the house floor was recorded. The motor coordination score was calculated as 3 points, 2 points, or 1 point for each rat that could completely pass the upper half within 3 s, 6 s or longer than 6 s, respectively (Zhao et al. 2019).

#### Open field test

Open field is an experimental test that has been widely used to assess the exploratory behavior and general activity of rats, whether hypoactive, hyperactive, or lost their motility. The test is based on the rats' natural aversion to brightly lit open areas and their anticipation for any potential threat (Hall 1932). The open field arena was made of a wooden box (40 × 40 in diameter) with 40 cm height, red walls to prevent the animal from escaping, and a white wooden board floor divided into 16 equal squares (10 X 10 cm^2^) by lines marked by a black color. The test was performed in a quiet place,; away from any disturbance, since increasing the levels of anxiety might lead to less activity and a preference to stay close to the walls of the field, while decreasing anxiety might increase the exploratory behavior of rats. During the test, each rat was gently placed in one of the arena corners and allowed to explore it for 3 min. For each rat, the following behavioral variables were measured: (i) ambulation frequency, (ii) frequency of rearing behavior, (iii) grooming frequency and (iv) latency time (s) from the time of dropping the rat into the field arena until starting its move.

### Brain tissue collection

Treated rats, except those used for cognitive–behavioral analysis, were euthanized via cervical decapitation, and their brains were excised. Each rat's brain was cut longitudinally into right and left halves, where the right brain halves were stored at – 20 ºC to be used later for preparing brain tissue homogenate. The left halves were washed with ice-cold saline, blotted to dryness, and then placed in 10% formalin for histopathological and immunohistochemical examination.

### Brain tissue homogenate preparation

The brain tissues of each left half were divided into four parts. One part was homogenized in 1 mL cold PBS with adding 0.16 mg heparin/mL to remove any red blood clots. Then, the homogenate was centrifuged at 4000×*g* for 15 min and the clear supernatant was collected to be used for assessing the oxidant/antioxidant status. The second part was rinsed in ice-cold PBS, homogenized in 10 mL lysis buffer, centrifuged at 10,000×*g* for 5 min, and then the supernatant was gathered for determining the concentrations of pro- and neuroinflammatory biomarkers. The third one was lysed using 1 mL of tissue protein extraction reagent (T-PER) and homogenized. Then, the lysate solution was centrifuged at 10,000×*g* for 5 min to pellet tissue debris. The supernatant was collected and the protein concentration was measured using BCA assay for determining AChE activity. The last part was immersed in liquid nitrogen to snap freeze, homogenized as 100 mg tissue/mL cold PBS, centrifuged at 12,000×*g* for 15 min, and then the supernatant was stored at -80 °C for western blotting assay of the MAPK pathway.

### Biochemical estimation of brain oxidant/antioxidant status

The concentrations of MDA (nmoL/mg tissue) and NO (µmol/g tissue), as well as TAC (μmol/mg tissue) in brain tissue samples were determined at the absorbance of 534 nm, 540 nm, and 505 nm, respectively (Koracevic et al. 2001; Ohkawa et al. 1979), using a DLAB SP-UV1100 Spectrophotometer (MediLab Tech co., USA).

### Biochemical determination of neuroprotective and inflammatory biomarkers

According to the manufacturer’s instructions supplied by ELISA kits, the brain concentrations of IL-10, GDNF, Dyn, IFN-γ, TNFα, and IL-1β were determined as (pg/g tissue) utilizing the Automated Microplate Elisa Reader (BioTek Instruments, Inc., USA).

### Colorimetric assay of AChE activity

The activity of AChE was measured at 570 nm and expressed as nmol/min/μg protein, based on the instructions provided by the kit.

### Western immunoblot assay of MAPK signaling pathway in brain tissue

A protein assay using bicinchoninic acid (BCA) reagent kit was performed to determine the protein content of each brain tissue sample. Proteins (20 µg) were run on the SDS-PAGE gel (4–12%) and subsequently moved out into PVDF membranes. The membranes were blocked with a blocking solution (5% skim milk in TBS and 0.05% Tween 20) for 1 h at room temperature and then incubated overnight at 4 °C with the targeted primary antibodies of 1:1000 dilution, including: anti-ERK1/2, anti-pERK1/2, anti-JNK, anti-p-JNK, anti-p38MAPK, anti-p-p38MAPK, and anti-β-actin (as an internal control). After that, blotting membranes were washed three times and thence incubated with IgG-horseradish peroxidase-conjugated secondary antibodies for 2 h at 37 °C. The relative level of target protein expression was determined.

### Histopathological investigation

Brain tissue samples were primarily fixed in formalin 10% (neutral-buffered) for at least 24 h. Then, samples were trimmed, processed, sectioned using manual microtome, stained with hematoxylin–eosin (HE) stain, inspected with light microscope, and captured using Olympus fixed camera (Jensen 2008).

### Immunohistochemical analysis of EGFR and GFAP

Representative tissue samples from brain tissue were collected from all experimental groups and subjected to immunohistochemistry staining with EGFR and GFAP primary antibodies. Immunohistochemical protocol was conducted as previously reported by Kim et al. (2016). Antigen retrieval was applied on brain tissue sections by heating. Then, the sections were incubated overnight with primary antibodies of EGFR and GFAP rabbit polyclonal antibody (diluted 1:200). Sections were washed with PBS to get rid of excess unbound antibodies and incubated with anti-rabbit IgG HRP antibody for 2 h. That was followed by another washing step and then by DAB staining and counterstaining with hematoxylin. Positive immunoreactivity was indicated by the presence of brown color. The intensity of immunoreactivity was graded as no staining, mild, moderate, or strong staining intensity.

### Statistical analysis

In the current investigation, the sample size was determined using the method proposed by Festing and Altman (2002) to determine the degree of freedom in ANOVA (E). E should be between 10 and 20 to increase the degree of significance. Simultaneously, the lowest and maximal number per group were assessed based on Arifin and Zahiruddin (2017).

For checking the continuous variables, both normality and homogeneity tests were performed using the Statistical Package for the Social Sciences (SPSS, Chicago, IL). One-way analysis of variance (ANOVA) was used to analyze the statistical significance between means, using a Tukey's honestly significant difference (HSD) post hoc test, if necessary for multiple comparisons.. For non-normal distributed data, the Kruskal–Wallis test followed by Dunn`s test was fulfilled. Data were expressed as mean ± SD, considering *P* < 0.05 as statistically significant.

## Results

### Effect of silymarin on the body weight of rats exposed to diacetyl

Our findings demonstrated that there was no significant difference in body weight across the experimental groups from the start to the completion of our study, except the rats of DA2 group that showed a marked decline in their body weights as compared to the control group at the end of week 4 (*P* < 0.05; Table 1).

**Table 1:** Effect of silymarin on the body weight of diacetyl-induced neuroinflammation rat model

Animal group	Mean body weight (g)				
	0 week	1st week	2nd week	3rd week	4th week
Control (C)	126.38 ± 3.42	157.38 ± 13.41	194.13 ± 12.98	234.13 ± 12.98	267.00 ± 14.36
Diacetyl-15 (DA1)	126.88 ± 4.52	146.75 ± 12.99	181.25 ± 11.06	–	–
Diacetyl-30 (DA2)	126.75 ± 5.18	144.88 ± 11.49	178.63 ± 12.55	220.93 ± 7.37	244.18 ± 7.41 ^a^
Silymarin (Sily)	126.00 ± 4.07	157.25 ± 15.60	194.75 ± 12.48	233.50 ± 11.53	267.50 ± 11.30
Protected (P)	126.50 ± 5.08	155.75 ± 10.98	193.13 ± 11.91	232.38 ± 8.83	265.75 ± 13.45
Treated-15 (Tl)	126.13 ± 4.32	154.25 ± 18.52	191.00 ± 12.08	230.25 ± 7.48	263.13 ± 9.46
Treated-30 (T2)	126.62 ± 3.78	150.50 ± 15.18	188.75 ± 11.45	229.25 ± 8.29	253.38 ± 11.24

Rats were orally treated once every day either with sterile distilled water (DW) as a vehicle (0.05 mL/kg/day; C group) for 15 days; diacetyl (DA) (100 mg/kg/day; DA1and DA2 group) for 15 and 30 days, respectively; silymarin (Sily) (50 mg/kg/day; Sily group) for 15 days; Sily 1 h before DA for 15 days (P group); DA for 15 days replaced by Sily at day 16 for another 15 days (T1 group) or DA for 15 days followed by Sily for the next 15 days parallel with DA administration (T2 group)

Non-significant change was detected in the body weight between all experimental groups, except at the 4th week. Data were expressed as means ± SD. Significant difference vs. ^a^ control (C), using one-way ANOVA followed by Tukey's honestly significant difference (HSD) post hoc; n = 8 per group

### Effect of silymarin on cognitive and behavioral function tests in a rat model of diacetyl-induced neuroinflammation

**Fig. 1:**
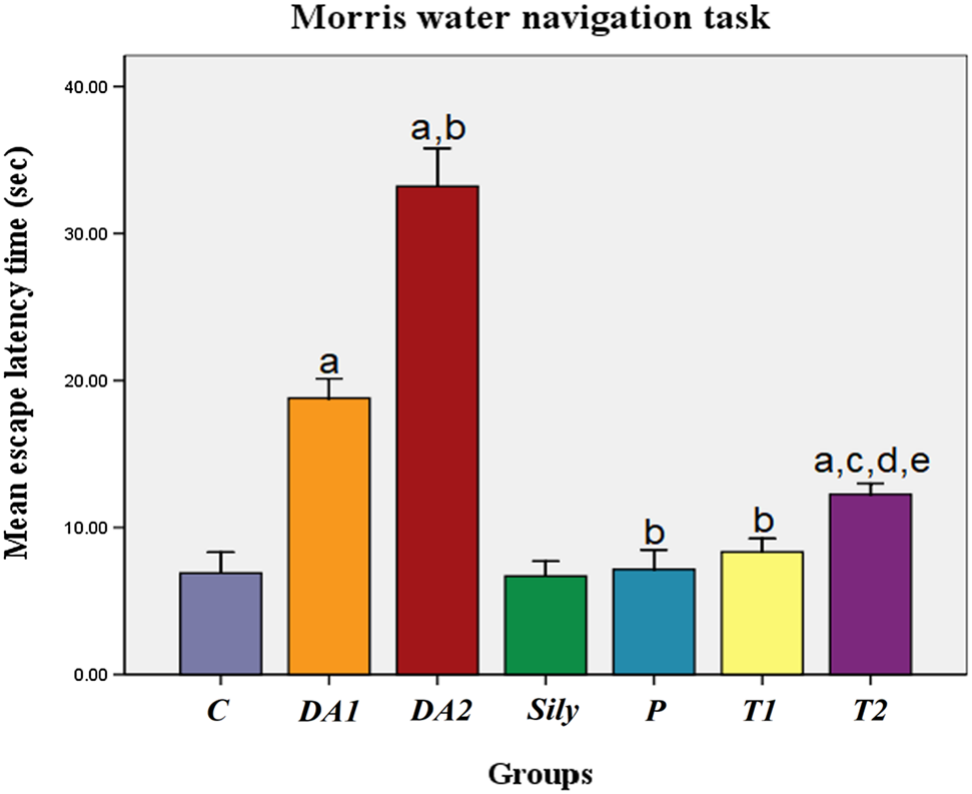
Effect of silymarin on Morris water navigation task in a rat model of diacetyl-induced neuroinflammation. Rats were orally treated once every day either with sterile distilled water (DW) as a vehicle (0.05 mL/kg/day; C group) for 15 days; diacetyl (DA) (100 mg/kg/day; DA1and DA2 group) for 15 and 30 days, respectively; Silymarin (Sily) (50 mg/kg/day; Sily group) for 15 days; Sily 1 h before DA for 15 days (P group); DA for 15 days replaced by Sily at day 16 for another 15 days (T1 group) or DA for 15 days followed by Sily for the next 15 days parallel with DA administration (T2 group). The cognitive performance of rats was expressed by recording the escape latency time (s). Data were expressed as means ± SD; significant difference vs. ^a^control (C), ^b^diacetyl-15 (DA1), ^c^diacetyl-30 (DA2), ^d^Silymarin (Sily) or ^e^treated-15 (T1) groups, each at *p* < 0.05, using one-way ANOVA followed by Tukey's honestly significant difference (HSD) post hoc; *n* = 4 per group

**Fig. 2:**
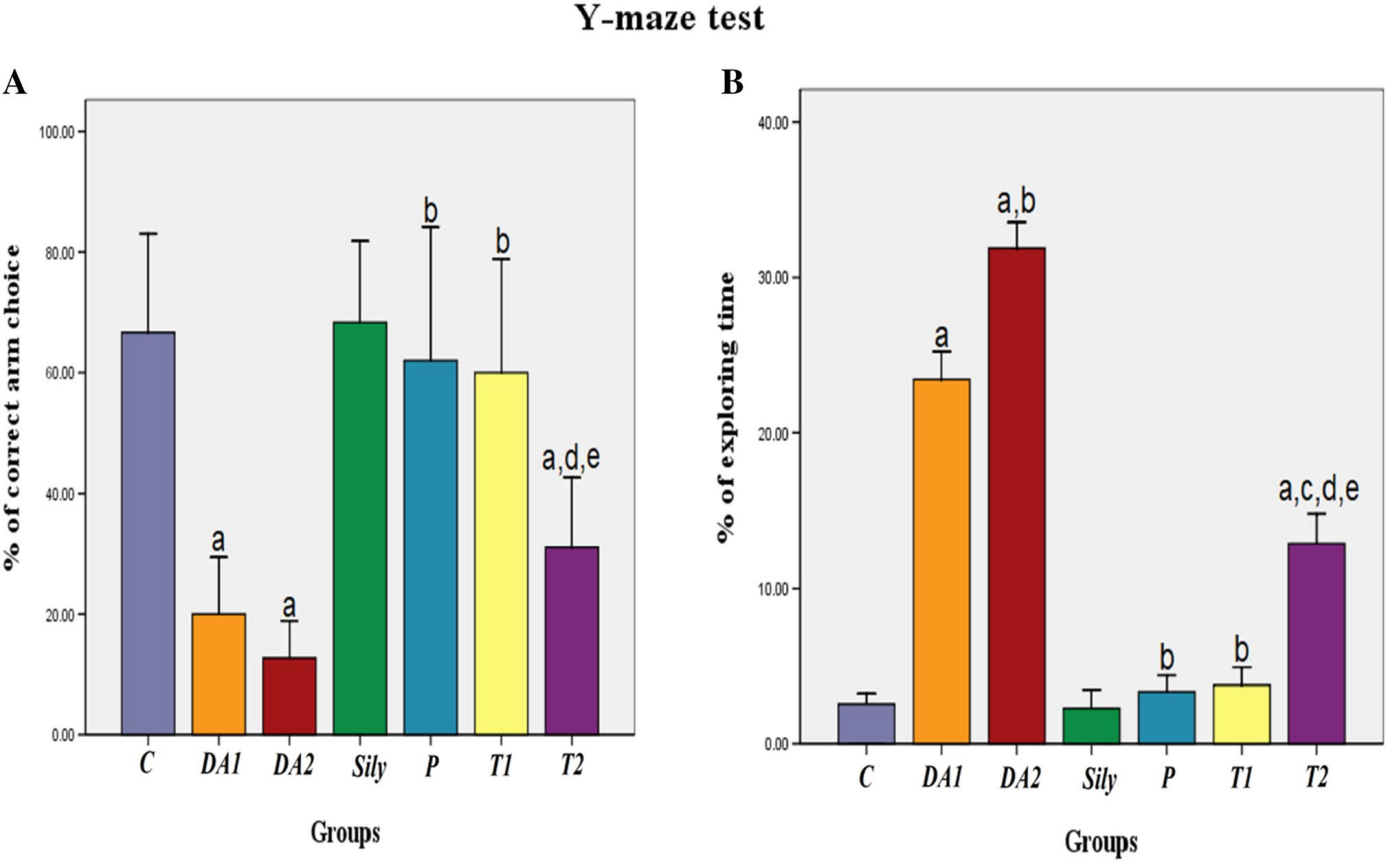
Effect of silymarin on Y-maze test in a rat model of diacetyl-induced neuroinflammation. Rats were orally treated once every day either with sterile distilled water (DW) as a vehicle (0.05 mL/kg/day; C group) for 15 days; diacetyl (DA) (100 mg/kg/day; DA1and DA2 group) for 15 and 30 days, respectively; Silymarin (Sily) (50 mg/kg/day; Sily group) for 15 days; Sily 1 h before DA for 15 days (P group); DA for 15 days replaced by Sily at day 16 for another 15 days (T1 group) or DA for 15 days followed by Sily for the next 15 days parallel with DA administration (T2 group). Recognition memory was assessed by calculating (A) % of correct arm choice and (B) % of exploring time. Data were expressed as means ± SD. Significant difference vs. ^a^control (C), ^b^diacetyl-15 (DA1), ^c^diacetyl-30 (DA2), ^d^silymarin (Sily) or ^e^treated-15 (T1) groups, each at *p* < 0.05, using one-way ANOVA followed by Tukey's honestly significant difference (HSD) post hoc; *n* = 4 per group

**Fig. 3:**
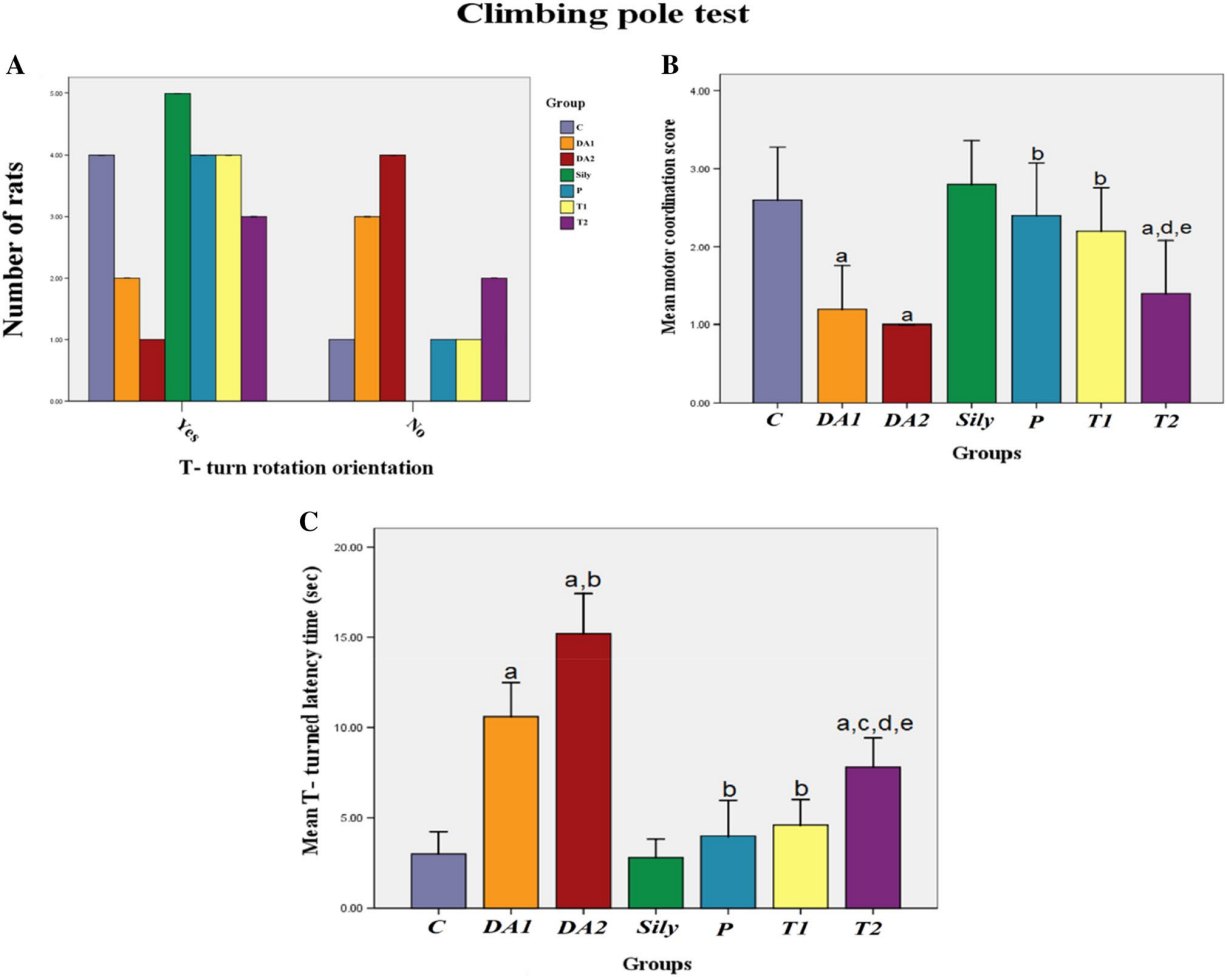
Effect of Silymarin on climbing pole test in a rat model of diacetyl-induced neuroinflammation. Rats were orally treated once every day either with sterile distilled water (DW) as a vehicle (0.05 mL/kg/day; C group) for 15 days; diacetyl (DA) (100 mg/kg/day; DA1and DA2 group) for 15 and 30 days, respectively; Silymarin (Sily) (50 mg/kg/day; Sily group) for 15 days; Sily 1 h before DA for 15 days (P group); DA for 15 days replaced by Sily at day 16 for another 15 days (T1 group) or DA for 15 days followed by Sily for the next 15 days parallel with DA administration (T2 group). Motor coordination function was determined by measuring (**A**) the number of T-turn rotated rats, (**B**) motor coordination score, and (**C**) T-turned latency time (s). Data were expressed as means ± SD; significant difference vs. ^a^control (C), ^b^diacetyl-15 (DA1), ^c^diacetyl-30 (DA2), ^d^Silymarin (Sily) or ^e^treated-15 (T1) groups, each at p < 0.05, using Pearson's Chi-squared test, Kruskal––Wallis test followed by Dunn`s test, and one-way ANOVA followed by Tukey's honestly significant difference (HSD) post hoc for (**A**), (**B**), and (**C**), respectively; *n* = 4 per group

To detect the effect of Sily on the cognitive and behavioral function, Morris water navigation task, Y-maze, climbing pole, and open field tests were applied. As compared to the control (C) group, we found that exposing rats to DA either for 15 or 30 days decreased their ability: (i) to find the correct path of the invisible submerged platform required for climbing and escaping: inferred by a significant increase in their escape latency time, (ii) to explore the arm of food reward: resulted in a significant decrease in the % of correct arm choice with a marked increase in the % of exploring time, (iii) to orient themselves, grasp the rod, and maneuver till descending downward: assessed by a decline in the number of T-turn rotated rats and in the motor coordination score with an observed increase in the T-turned latency time, and (iv) to move away from brightly lit open areas and to anticipate any potential threat: indicated by a decrease in the ambulation frequency and in the frequency of rearing behavior with a significant increase in the grooming frequency and latency time. On the other hand, both P and T1 groups as well as the T2 group showed a significant decrease in the escape latency time, % of exploring time, T-turned latency time, grooming frequency, and latency time of open field test with significant elevation in the % of correct arm choice, number of T-turn rotated rats, motor coordination score, ambulation frequency, and frequency of rearing behavior versus the DA1 and DA2 groups, respectively. The T2 group also produced a significant increase in the escape latency time, % of exploring time, T-turned latency time, grooming frequency, and latency time of open field test with significant decrease in the % of correct arm choice, number of T-turn rotated rats, motor coordination score, ambulation frequency, and frequency of rearing behavior as compared to the C, Sily, and T1 groups (*P* < 0.05; Fig. 1, Figs. 2A, B, and 3A, B & C, Table 2).

**Table 2:** Effect of silymarin on open field test in a rat model of diacetyl-induced neuroinflammation

Animal groups	Mean ambulation frequency	Mean frequency of rearing behavior	Mean grooming frequency	Mean latency time (s)
C	29.0 ± 3.16	24.8 ± 2.39	3.0 ± 1.00	30.6 ± 3.05
DA1	6.6 ± 2.70 ^a^	12.0 ± 1.58 ^a^	12.0 ± 1.58 ^a^	60.2 ± 4.38 ^a^
DA2	2.8 ± 0.84^a,b^	5.00 ± 1.57^a,b^	16.2 ± 1.30^a,b^	7.66 ± 2.88^a,b^
Sily	31.0 ± 2.92	26.2 ± 1.92	2.8 ± 0.84	28.6 ± 2.07
P	27.6 ± 2.7 ^b^	23.2 ± 1.90 ^b^	3.4 ± 1.14 ^b^	33 ± 2.74 ^b^
T1	26.2 ± 1.9 ^b^	22.4 ± 1.52 ^b^	3.6 ± 1.11 ^b^	36 ± 2.92 ^b^
T2	13.2 ± 2.86 ^a,c,d,e^	16.0 ± 2.24 ^a,c,d,e^	7.0 ± 2.74 ^a,c,d,e^	44 ± 3.39 ^a,c,d,e^

Rats were orally treated once every day either with sterile distilled water (DW) as a vehicle (0.05 mL/kg/day; C group) for 15 days; diacetyl (DA) (100 mg/kg/day; DA1and DA2 group) for 15 and 30 days, respectively; silymarin (Sily) (50 mg/kg/day; Sily group) for 15 days; Sily 1 h before DA for 15 days (P group); DA for 15 days replaced by Sily at day 16th for another 15 days (T1 group) or DA for 15 days followed by Sily for next 15 days parallel with DA administration (T2 group). Significant differences were detected between all experimental groups (*P* < 0.05). Data were expressed as means ± SD vs

^a^Control (C)

^b^Diacetyl-15 (DA1)

^c^Diacetyl-30 (DA2)

^d^Silymarin (Sily) or ^e^ treated-15 (T1) groups, each at *p* < 0.05, using one-way ANOVA followed by Tukey's honestly significant difference (HSD) post hoc; *n* = 4 per group

### Effect of silymarin on brain oxidant/antioxidant status in a rat model of diacetyl-induced neuroinflammation

**Fig. 4:**
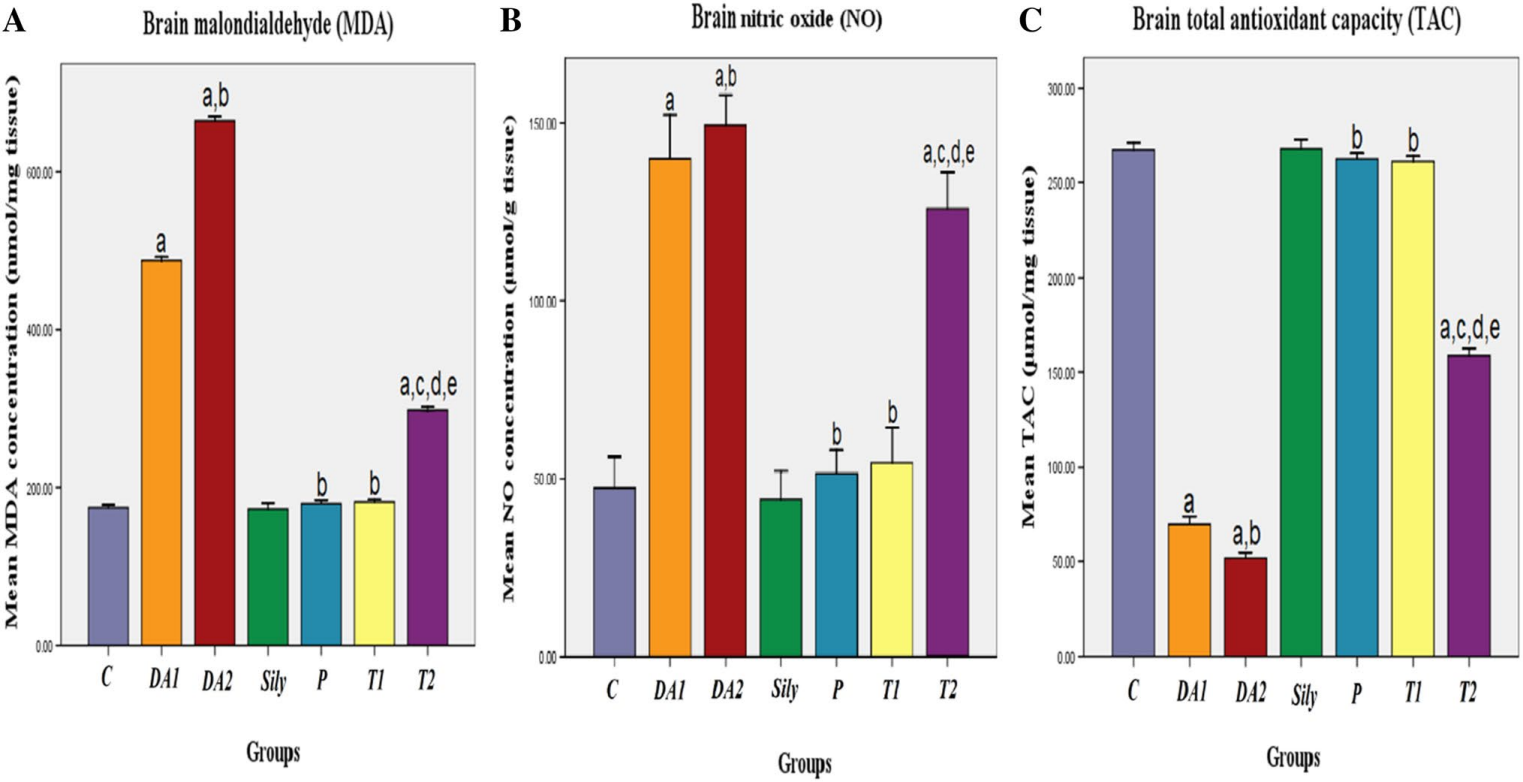
Effect of silymarin on brain oxidant/antioxidant status in a rat model of diacetyl-induced neuroinflammation. Rats were orally treated once every day either with sterile distilled water (DW) as a vehicle (0.05 mL/kg/day; C group) for 15 days; diacetyl (DA) (100 mg/kg/day; DA1and DA2 group) for 15 and 30 days, respectively; Silymarin (Sily) (50 mg/kg/day; Sily group) for 15 days; Sily 1 h before DA for 15 days (P group); DA for 15 days replaced by Sily at day 16 for another 15 days (T1 group) or DA for 15 days followed by Sily for the next 15 days parallel with DA administration (T2 group). The brain oxidant/antioxidant parameters (**A**) malondialdehyde (MDA) (nmol/mg tissue), (**B**) nitric oxide (NO) (µmol/g tissue), and (**C**) total antioxidant capacity (TAC) (μmol/ mg tissue) were evaluated. Data were expressed as means ± SD; significant difference vs. ^a^control (C), ^b^diacetyl-15 (DA1), ^c^diacetyl-30 (DA2), ^d^silymarin (Sily) or ^e^treated-15 (T1) groups, each at *p* < 0.05, using one-way ANOVA followed by Tukey's honestly significant difference (HSD) post hoc; *n* = 4 per group

To recognize the antioxidant role of Sily, MDA and NO concentrations as well as TAC were determined in brain tissues. DA-intoxicated rats either for 15 or 30 days showed a significant increase in the levels of MDA and NO with a marked decrease in TAC as compared to the C group. On the contrary, the P and T1 groups as well as the T2 group produced a significant decrease in the brain MDA and NO levels with an increase in TAC against the DA1 and DA2 groups, respectively. Furthermore, a significant increase in MDA and NO levels with a decrease in TAC was detected on comparing the T2 group to the C, Sily, and T1 groups (*P* < 0.05; Fig. 4A, B, C).

### Effect of silymarin on brain levels of neuroprotective biomarkers in a rat model of diacetyl-induced neuroinflammation

**Fig. 5:**
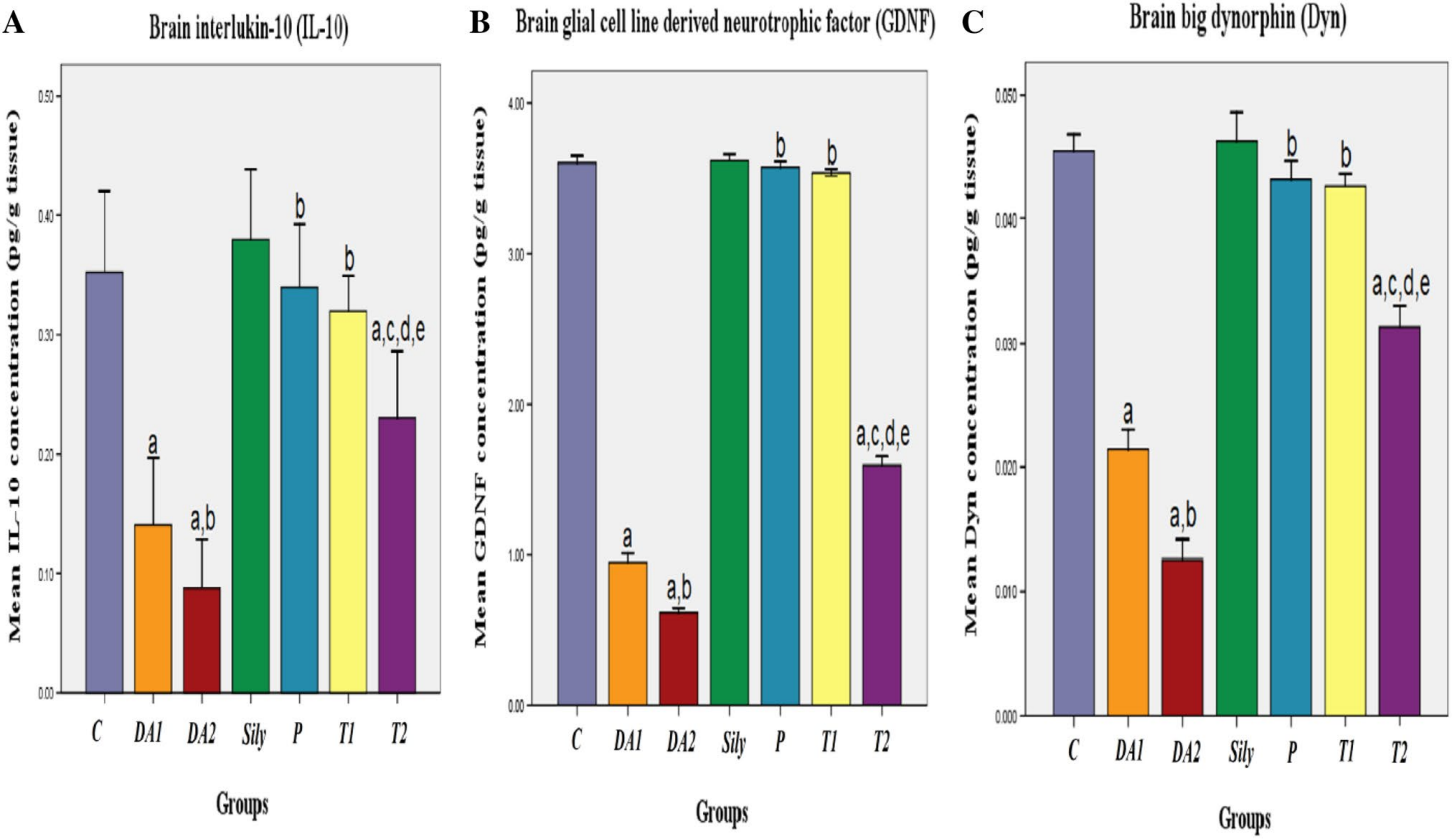
Effect of silymarin on brain levels of neuroprotective biomarkers in a rat model of diacetyl-induced neuroinflammation. Rats were orally treated once every day either with sterile distilled water (DW) as a vehicle (0.05 mL/kg/day; C group) for 15 days; diacetyl (DA) (100 mg/kg/day; DA1and DA2 group) for 15 and 30 days, respectively; Silymarin (Sily) (50 mg/kg/day; Sily group) for 15 days; Sily 1 h before DA for 15 days (P group); DA for 15 days replaced by Sily at day 16 for another 15 days (T1 group) or DA for 15 days followed by Sily for the next 15 days parallel with DA administration (T2 group). The brain levels of anti-neuroinflammatory biomarkers (**A**) interleukin-10 (IL-10) (pg/g tissue), (**B**) glial cell line derived neurotrophic factor (GDNF) (pg/g tissue), and (**C**) big dynorphin (Dyn) (pg/g tissue) were measured. Data were expressed as means ± SD; significant difference vs. ^a^control (C), ^b^diacetyl-15 (DA1), ^c^diacetyl-30 (DA2), ^d^Silymarin (Sily) or ^e^treated-15 (T1) groups, each at *p* < 0.05, using one-way ANOVA followed by Tukey's honestly significant difference (HSD) post hoc; n = 4 per group

To detect the neuroprotective effect of Sily, the brain levels of IL-10, GDNF, and Dyn were assessed. The results showed that exposing rats to DA either for 15 or 30 days significantly decreased IL-10, GDNF, and Dyn concentrations as compared to the C group. On the contrary, the P and T1 groups as well as the T2 group presented a significant increase in IL-10, GDNF, and Dyn versus the DA1 and DA2 groups, respectively. Moreover, a significant decrease was reported in IL-10, GDNF, and Dyn levels of the T2 group when compared with the C, Sily, and T1 groups (**P < 0.05; **Fig. 5A, B).

### Effect of silymarin on the brain levels of inflammatory cytokines in a rat model of diacetyl-induced neuroinflammation

**Fig. 6:**
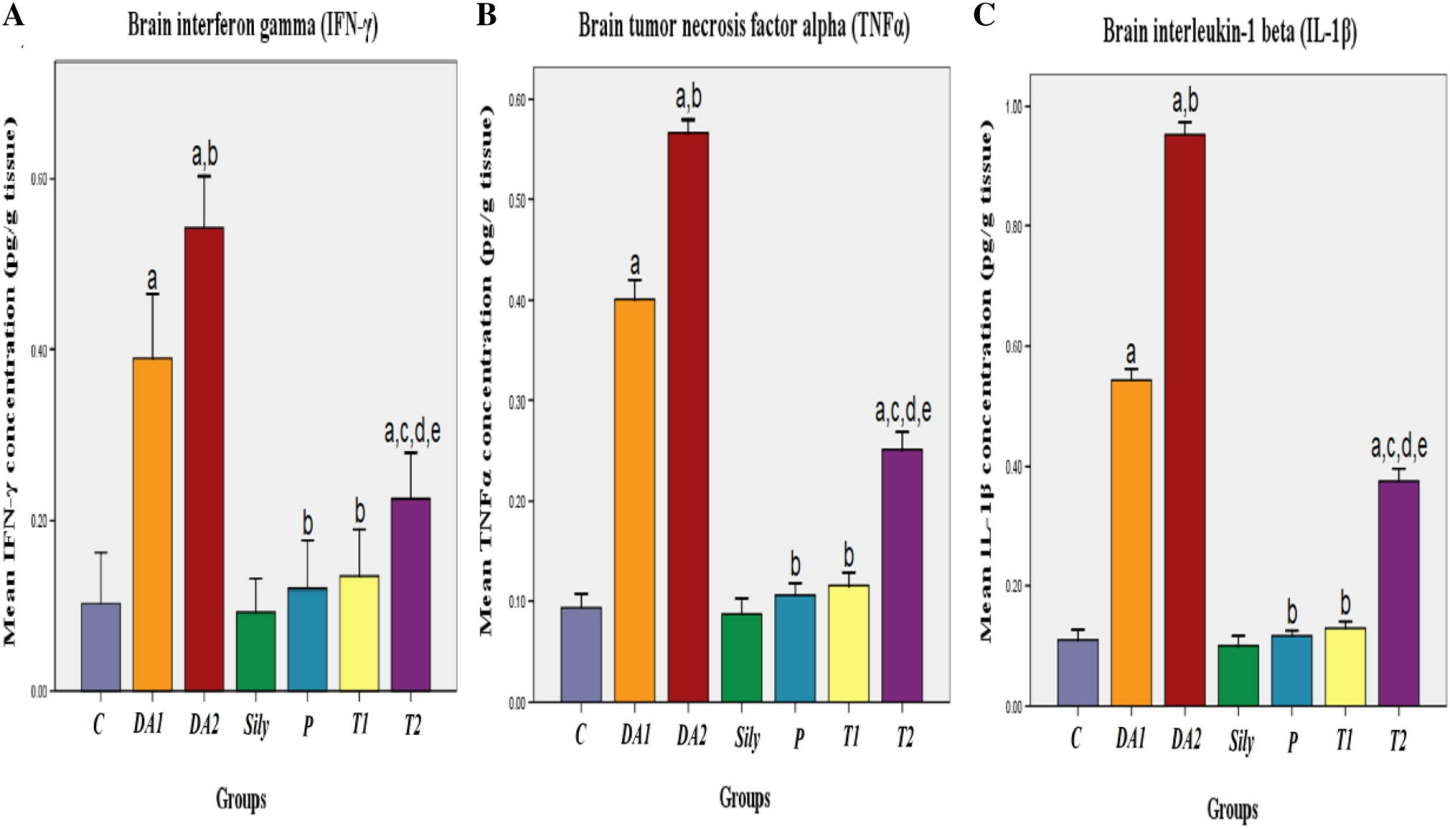
Effect of silymarin on brain levels of inflammatory cytokines in a rat model of diacetyl-induced neuroinflammation. Rats were orally treated once every day either with sterile distilled water (DW) as a vehicle (0.05 mL/kg/day; C group) for 15 days; diacetyl (DA) (100 mg/kg/day; DA1and DA2 group) for 15 and 30 days, respectively; Silymarin (Sily) (50 mg/kg/day; Sily group) for 15 days; Sily 1 h before DA for 15 days (P group); DA for 15 days replaced by Sily at day 16 for another 15 days (T1 group) or DA for 15 days followed by Sily for the next 15 days parallel with DA administration (T2 group). The brain concentrations of pro-inflammatory cytokines (**A**) interferon gamma (IFN-γ) (pg/g tissue), (**B**) tumor necrosis factor alpha (TNFα) (pg/g tissue), and (**C**) interleukin-1 beta (IL-1β) (pg/g tissue) were estimated. Data were expressed as means ± SD; significant difference vs. ^a^control (C), ^b^diacetyl-15 (DA1), ^c^diacetyl-30 (DA2), ^d^silymarin (Sily) or ^e^treated-15 (T1) groups, each at *p* < 0.05, using one-way ANOVA followed by Tukey's honestly significant difference (HSD) post hoc; *n* = 4 per group

To evaluate the anti-inflammatory properties of Sily against cytokines produced in the brain tissues, we analyzed the levels of IFN-γ, TNFα, and IL-1β. It was found that DA-exposed rats either for 15 or 30 days showed a significant increase in levels of IFN-γ, TNFα, and IL-1β versus C group. On the other hand, the P and T1 groups as well as the T2 group displayed a marked decrease in the brain levels of IFN-γ, TNFα, and IL-1β as compared to the DA1 and DA2 groups, respectively. Additionally, there was a significant increase in the levels of IFN-γ, TNFα, and IL-1β of the T2 group compared with the C, Sily, and T1 groups (*P* < 0.05; Fig. 6A, B).

### Effect of silymarin on AChE activity in a rat model of diacetyl-induced neuroinflammation

**Fig. 7:**
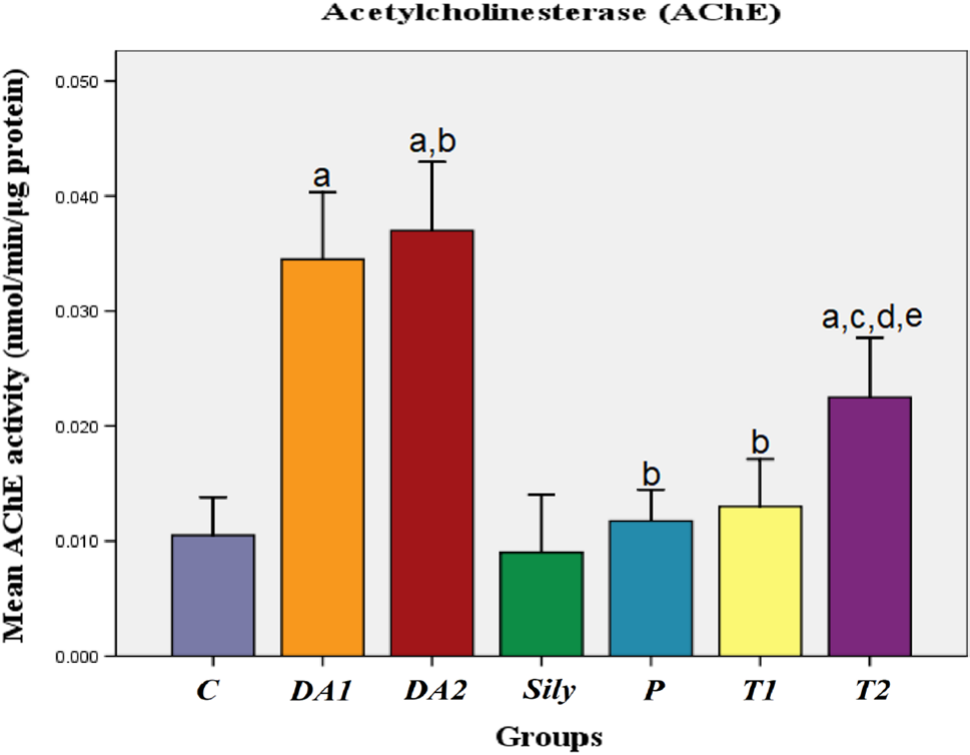
Effect of silymarin on AChE activity in a rat model of diacetyl-induced neuroinflammation. Rats were orally treated once every day either with sterile distilled water (DW) as a vehicle (0.05 mL/kg/day; C group) for 15 days; diacetyl (DA) (100 mg/kg/day; DA1and DA2 group) for 15 and 30 days, respectively; Silymarin (Sily) (50 mg/kg/day; Sily group) for 15 days; Sily 1 h before DA for 15 days (P group); DA for 15 days replaced by Sily at day 16 for another 15 days (T1 group) or DA for 15 days followed by Sily for the next 15 days parallel with DA administration (T2 group). The AChE activity (nmol/min/μg protein) was measured. Data were expressed as means ± SD; significant difference vs.^a^control (C), ^b^diacetyl-15 (DA1), ^c^diacetyl-30 (DA2), ^d^silymarin (Sily) or ^e^treated-15 (T1) groups, each at *p* < 0.05; using one-way ANOVA followed by Tukey's honestly significant difference (HSD) post hoc; *n* = 4 per group

In comparison to the C group, rats that received DA for 15 or 30 days exhibited a considerable increase in AChE activity. The activity of AChE was also significantly reduced in the P and T1 groups, as well as the T2 group, as compared to both the DA1 and DA2 groups. Furthermore, when comparing the T2 group to the C, Sily, and T1 groups, a substantial increase in AChE activity was discovered (*P* < 0.05; Fig. 7).

### Effect of silymarin on brain MAPK signaling pathway in a rat model of diacetyl-induced neuroinflammation

**Fig. 8:**
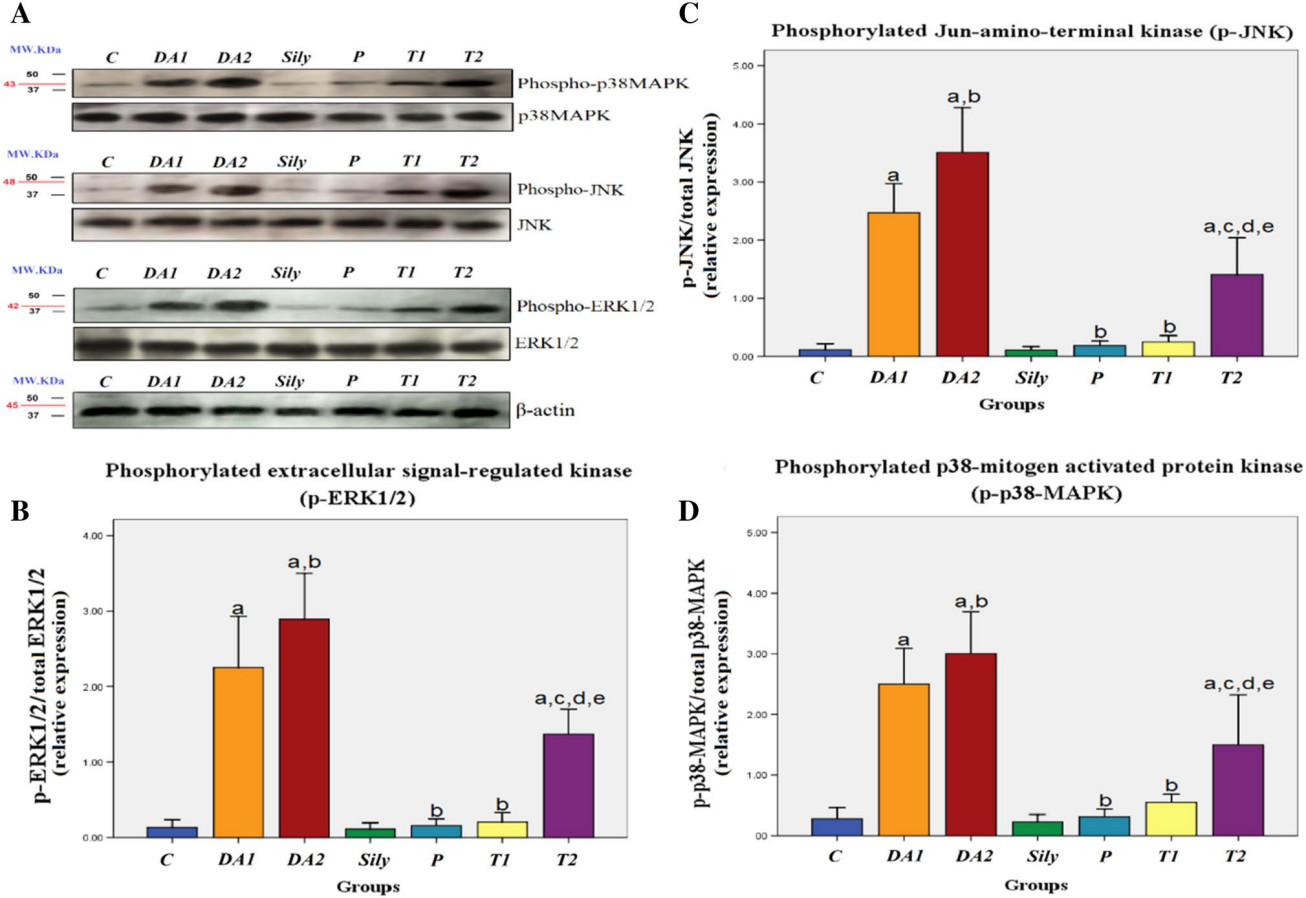
Effect of silymarin on brain MAPK signaling pathway in a rat model of diacetyl-induced neuroinflammation. Rats were orally treated once every day either with sterile distilled water (DW) as a vehicle (0.05 mL/kg/day; C group) for 15 days; diacetyl (DA) (100 mg/kg/day; DA1and DA2 group) for 15 and 30 days, respectively; Silymarin (Sily) (50 mg/kg/day; Sily group) for 15 days; Sily 1 h before DA for 15 days (P group); DA for 15 days replaced by Sily at day 16 for another 15 days (T1 group) or DA for 15 days followed by Sily for the next 15 days parallel with DA administration (T2 group). Western blotting was performed for (**A**) ERK1/2, phospho-ERK1/2, JNK, phospho-JNK, p38-MAPK, phospho-p38-MAPK, and β-actin. The regulation of MAPK signaling was assayed by the ratio of (**B**) phosphorylated-ERK1/2/total ERK1/2/ERK1/2, (**C**) phosphorylated JNK/total JNK, and (**D**) phosphorylated p38/total p38. β-Actin was used as an internal control and data was expressed as mean ± S.D; significant difference vs. ^a^control (C), ^b^diacetyl-15 (DA1), ^c^diacetyl-30 (DA2), ^d^Silymarin (Sily) or ^e^treated-15 (T1) groups, each at *p* < 0.05, using one-way ANOVA followed by Tukey's honestly significant difference (HSD) post hoc; *n* = 3 per group

Our data showed that DA-exposed rats either for 15 or 30 days revealed a significant increase in the expression ratio of p-ERK1/2, p-JNK, and p-p38-MAPK as compared to the C group. On the contrary, the P and T1 groups as well as the T2 group showed a significant decrease in the expression ratio of p-ERK1/2, p-JNK, and p-p38-MAPK versus the DA1 and DA2 groups, respectively. There was also a significant increase in the relative expression of p-ERK1/2, p-JNK, and p-p38-MAPK of the T2 group when compared with the C, Sily, and T1 groups (*P* < 0.05; Fig. 8A, B, C & D).

### Histopathological effect of silymarin on the brain tissue of rat model for diacetyl-induced neuroinflammation

**Fig. 9:**
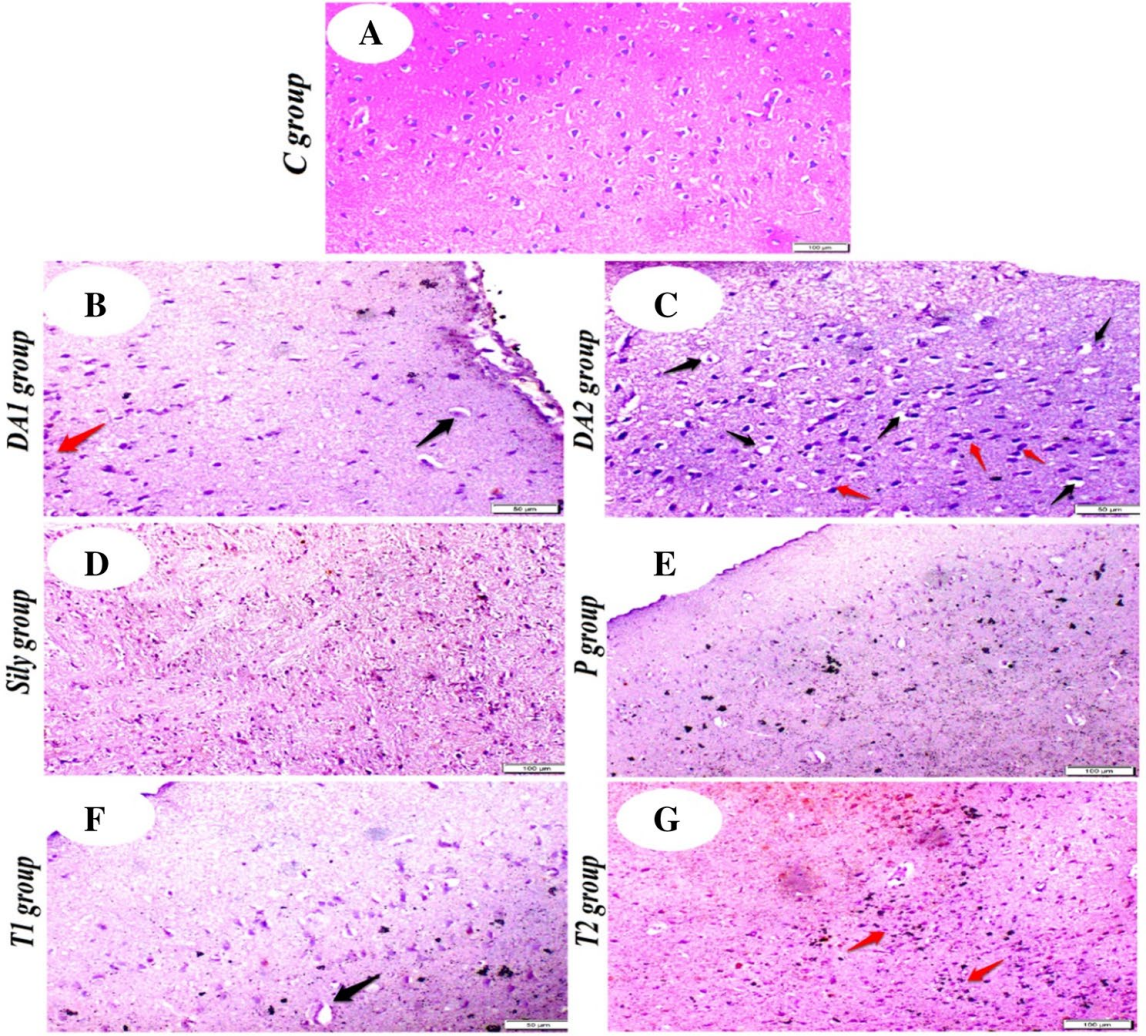
Photomicrographs showing the effect of silymarin on histopathological changes in the brain tissue of rat model for diacetyl-induced neuroinflammation (hematoxylin and eosin, H&E stain). **A** control (**C**) group, showing normal histological picture of the brain tissue, (**B**) diacetyl-15 (DA1) group, showing scattered neuronal edema (black arrow) and few aggregations of inflammatory cells (red arrow), (**C**) diacetyl-30 (DA2) group, showing severe neuronal edema (black arrows) and heavy infiltration of inflammatory cells within the brain tissue, (**D**) silymarin (Sily) group, showing normal histological picture of the brain tissue, (**E**) protected (P) group, showing normal histological picture of the brain and meninges, (**F**) treated-15 (T1) group, showing mild neuronal edema (black arrow) and (**G**) treated-30 (T2) group**,** showing focal aggregates of inflammatory cells in brain tissue (red arrows). Scale bars = 100 µm (**A**, **D**, **E** & **G** photos); 50 µm (B, C &F photos)

As compared to the C group (Fig. 9A), histopathologic examination of brain tissue in the rat model of DA-induced neuroinflammation revealed scattered neuronal edema with few aggregations of inflammatory cells in the DA1 group (Fig. 9B), while showing more severe neuronal edema and heavy infiltration of inflammatory cells in the DA2 group (Fig. 9C). On the other hand, both the Sily and P groups showed a normal histological picture of the brain tissue (Fig. 9D, E). At the same time, the T1 group showed less degree of inflammation with only mild neuronal edema (Fig. 9F). For the T2 group, focal aggregates of inflammatory cells in brain tissue were detected (Fig. 9G).

### Effect of silymarin on EGFR immunostaining in the brain tissue of rat model for diacetyl-induced neuroinflammation

**Fig. 10:**
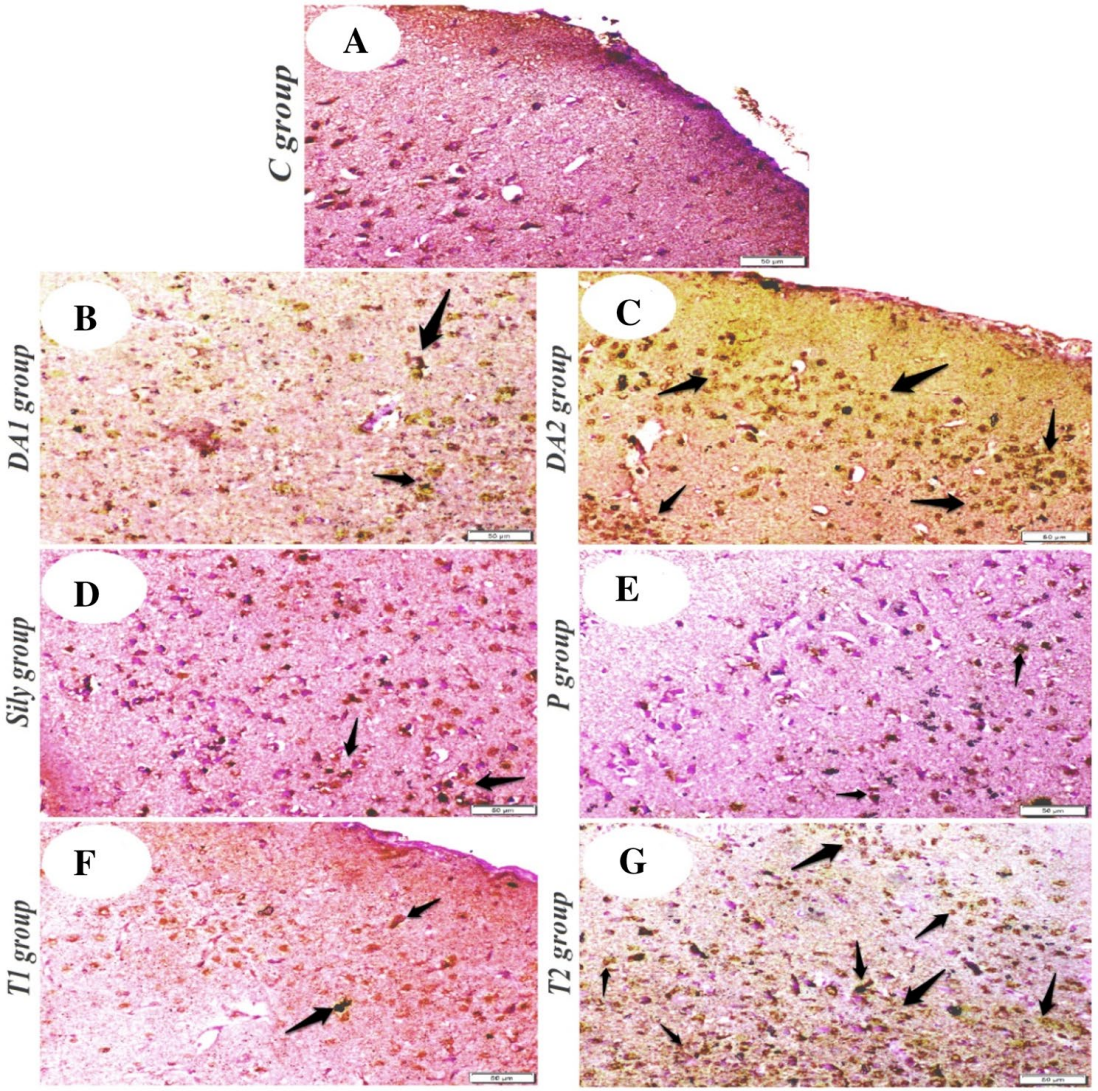
Photomicrographs showing the effect of silymarin on epidermal growth factor receptor (EGFR) immunostaining in the brain tissue of rat model for diacetyl-induced neuroinflammation determined (immunohistochemically; IHC). (**A**) control (C) group, showing no immunostaining with EGFR, (**B**) diacetyl-15 (DA1) group, showing positive immunoreactivity with moderate immunostaining (arrows), (**C**) diacetyl-30 (DA2) group, showing strong positive immunoreactivity, (**D**) silymarin (Sily) group, showing less neurons with positive immunoreactivity (arrows), (**E**) protected (P) group, showing few neurons with positive immunostaining (arrows), (**F**) treated-15 (T1) group, showing scattered positive immunostaining with weak intensity (arrows) and (**G**) treated-30 (T2) group, showing positive immunoreactivity with moderate immunostaining (arrows). Scale bars = 50 µm

The results showed that both C and Sily groups showed no immunoreactivity signals for EGFR (Fig. 10A, D). On the contrary, positive immunoreactivity with moderate immunostaining was observed in both the DA1 and T2 groups (Fig. 10B, G), while severe immunostaining was noted in DA2 (Fig. 10C). To a lesser degree, the P and T1 groups presented few scattered neurons with positive immunostaining (Fig. 10E, F).

### Effect of silymarin on GFAP immunostaining in the brain tissue of rat model for diacetyl-induced neuroinflammation

**Fig. 11:**
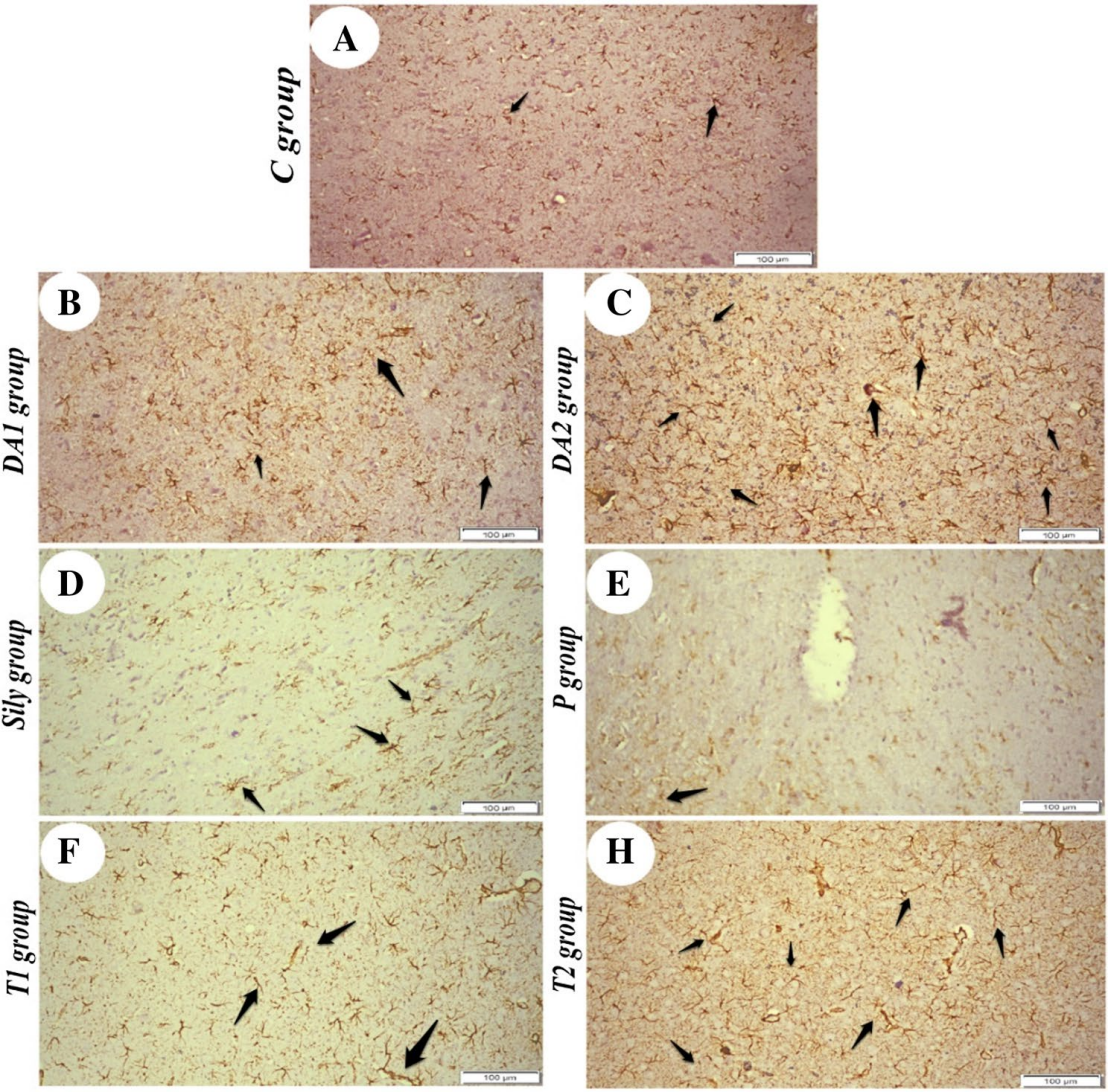
Photomicrographs showing the effect of silymarin on glial fibrillary acidic protein (GFAP) immunostaining in the brain tissue of rat model for diacetyl-induced neuroinflammation (immunohistochemically; IHC). (**A**) control (C) group, showing few scattered immunostained cells (arrows), (**B**) diacetyl-15 (DA1) group, showing positive immunostaining with moderate intensity (arrows), (**C**) diacetyl-30 (DA2) group, showing strong positive immunostaining (arrows), (**D**) silymarin (Sily) group, showing few scattered immunostained cells with very mild intensity (arrows), (**E**) protected (P) group, showing few scattered positive immunostaining (arrow), (**F**) treated-15 (T1) group, showing mild scattered positive immunostaining (arrows) and (**G**) treated-30 (T2) group, showing moderate intensity of immunostaining (arrows). Scale bars = 100 µm

According to the findings, the C and Sily groups exhibited few scattered immunostained cells for GFAP with very mild intensity (Fig. 11A, D). In an opposite manner, the DA1 and T2 groups showed moderate intensity of positive immunostaining (Fig. 11B, G), whereas DA2 displayed strong positive immunostaining (Fig. 11C). Both the P and T1 groups had a few mild scattered positive immunostaining (Fig. 11E, F).

## Discussion

Many toxicological studies have focused on the DA occupational effects on industrial workers' airways, neglecting the potentially detrimental consequences of DA intake with meals and drinks (Hubbs et al. 2019; Kreiss 2017). This leads us to the critical need for directing the attention of scientific research toward assessing the negative health impacts associated with DA oral delivery in consumers. Simultaneously, no investigation has addressed the idea that DA might harm the CNS and thereafter be a risk factor for neuroinflammation.

Since the pathological pathway by which DA may promote inflammation of the nervous tissue is still unclear, it was necessary to create an appropriate animal model representing systemic DA exposure to explain well the precise mechanisms of evoked neuronal inflammation within the brain or spinal cord and, hence,to screen and study potential neuroprotective agents such as silymarin (Sily). Sily, a widely available hepatoprotective medication, has been demonstrated to exert excellent protective and therapeutic functions against a variety of CNS disorders (Borah et al. 2013; Milić et al. 2013). This finding pushed us to explore the possible role that Sily may play in protecting and/or treating neuroinflammation induced by consuming DA in a rat model.

The results of the present study affirm that animals administered DA at a dosage 25 mg/kg/day either for 15 or 30 days showed a decline in their spatial learning, recognition memory, and motor coordination, in addition to the exploratory behavior and general activity as compared to the normal group, which is consistent with a previous research. The study showed that DA at the same dose (25 mg/kg/day) for 4 weeks might cause damage in both brain and testicular tissue (Bawazir 2016), although that dose was reported to be lower than that previously estimated as a no-effect dose (90 mg/kg/day) (Colley et al. 1969). Similarly, Jedlicka et al. had observed that animals treated with DA at higher dose (300 mg/kg/day) for 15 weeks exhibited behavioral abnormalities and had linked these changes to an imbalance between oxidants and antioxidants (Jedlicka et al. 2018).

Because of the uncontrolled acetylation, DA could cause changes in the structure and function of mitochondrial proteins, interfering with their stability and enzymatic activity (Jedlicka et al. 2018) and, therefore, leading to a rise in the ROS production and lessening the activities of antioxidant enzymes (Larsen et al. 2009). The same findings were recorded in the current study, since ingesting DA either for 15 or 30 days caused a disturbance in the brain oxidant/antioxidant status, as indicated by an increase in MDA level with a decrease in TAC.

Considering the DA-induced oxidative stress is an initial spark for producing neuronal inflammation and associated CNS malfunction, using an antioxidant agent with neuroprotective role such as Sily may be beneficial. The results of this study recorded that Sily (50 mg/kg/day) either with or after DA exposure could improve the consolidation of learning, memory, motor, and general activity in both Sily-protected and -treated rats, which corresponds with a recent research that showed that Sily supplementation with the same oral dose for 7 days protected rats from docetaxel-induced neurotoxicity by lowering oxidative stress, inflammation, and apoptosis (Yardım et al. 2021).

Being a flavonoid, Sily has the ability to functionally inhibit lipid oxidation and scavenge the produced free radicals in the brain, reducing therefore the induced oxidative stress (Moens et al. 2013; Yön et al. 2019). In this way, rats that received Sily could restore the brain oxidant/antioxidant balance through reducing the MDA level and enhancing TAC in brain tissues. The same was reported in a previous investigation which stated that giving Sily intraperitoneally at the same dose once a day for 8 days significantly decreased the MDA level in the vancomycin-induced nephrotoxicity rat model (Guo et al. 2019). Also, another study reported that using Sily (60 mg/kg/day) for 60 days counteracted the oxidative stress status in diabetic rats by increasing TAC (Anthony and Saleh 2013).

As mentioned, ROS overproduction had been shown to stimulate several neuroinflammatory signaling pathways, which could in turn lead to invigorating the main glial cells including microglia and astrocytes (Narita et al. 2018). In spite of the important functions that glia can provide in developing and maintaining BBB, ensuring brain homeostasis and protecting neurons, they may also have pernicious roles (Baeza et al. 2016). In CNS, microglia represent the primary active immunological defense that exists in a "resting" state. Once microglial cells have detected any change in the microenvironment caused either by infections, toxins, or injuries, they become "activated" (Solleiro-Villavicencio and Rivas-Arancibia 2018).

Typically, "active microglia" have been divided into two phenotypes: neurotoxic (M1) and neuroprotective (M2). The M1 phenotype "classical activation" is a pro-inflammatory state produced primarily as the first line of tissue defense in response to pro-inflammatory stimuli such as lipopolysaccharide (LPS) and interferon-γ (IFN-γ). Then, M1 induces inflammation and cytotoxicity by increasing the production of potentially cytotoxic molecules like inflammatory mediators (TNF-α, IL-1β, and NO) (Lively and Schlichter 2018), which in turn enhance AChE activity, elucidating the role of cholinergic system inhibition in mediating neuroinflammation (Tyagi et al. 2008). Conversely, resolving cytokines including IL-4 and IL-10 can counteract these inflammatory reactions by initiating the anti-inflammatory state (M2 phenotype). M2 is referred to as "alternative activation" since it is stimulated quickly to restore tissue homeostasis and to protect neurons from damage by secreting neuroprotective substances such as GDNF. Indeed, GDNF is an important neurotrophic protein in the brain, which is well known for promoting survival, development, and function of neurons. It has been detected to exert neurorestorative and neuroprotective roles in a variety of central and peripheral nervous system diseases (Arcuri et al. 2017).

It is also interesting to note that IL-10 may indirectly promote GDNF production by increasing the secretion of corticotropin releasing factor (CRF) (Li et al. 2014a), which in turn liberates endogenous dynorphin (Dyn) from the terminals of dynorphin-containing neurons (Song and Takemori 1992). Dyn is an opioid neuropeptide that works by modulating the activity of glial cells to reduce inflammatory reactions (Wang et al. 2012; Feng et al. 2013; Carniglia et al. 2017); however, its effectiveness as a neuroprotectant is still debatable. Lately, Dyn has been suggested to foster microglia polarization toward the M2 phenotype by suppressing the TLR4/NF-κB pathway (Liu et al. 2020) and, thereby, lead to a rise in GDNF level.

In the present investigation, producing more ROS as a result of continuous DA exposure for 15 or 30 days may promote IFN-γ secretion and, hence, overactivate the M1 phenotype microglia, leading to an increase in the release of inflammatory markers such as TNF-α, IL-1β, and NO as well as AChE activity with a decline in IL-10, GDNF, and Dyn levels. On the other hand, Sily has been found to possess neuroprotective effect through boosting IL-10 release (Darvishi-Khezri et al. 2021) and, consequently, formation of the M2-related GDNF either directly or indirectly via enhancing Dyn production.

Although there is currently no evidence on the effect of Sily on Dyn secretion, and this work may be the first to demonstrate a link between Sily treatment and enhanced Dyn production, Sily has been shown to inhibit ACTH secretion, restore negative glucocorticoid feedback, and then CRF level, which may account for its ability to simulate Dyn release (Vankrunkelsven et al. 2020). At the same time, an opposing investigation indicated that Sily might impede angiogenesis and thereby cause severe fibrosis in endometriotic-like lesions by down-regulating GDNF expression (Nahari and Razi 2018); however, this is in contrast to the previously stated preventative and curative capabilities of Sily.

Meanwhile, Sily could exert powerful anti-inflammatory activities through blocking IFN-γ-mediated M1 neurotoxic state production (Navabi et al. 2019; Zhao et al. 2021) and, consequently, lowering the levels of TNF-α, IL-1β, and NO (Haddadi et al. 2013), as well as AChE activity (Guo et al. 2019). As a result, giving Sily either before or with DA exposure in this study could effectively suppress the DA-induced inflammatory response within the brain.

Of interest, under the conditions of cellular stress and neuroinflammation, M1 phenotype microglial activation further activates the adjacent astrocytes into the A1 phenotype that will amplify the inflammatory response (Jurga et al. 2020) and this is accompanied by EGFR up-regulation to control reactive astrogliosis (Mitroshina et al. 2019). Similar results were observed in the histopathological and immunohistochemical investigation of brain tissues collected from rats that received DA either for 15 or 30 days in this study, where abnormal rise in the number of active astrocytes with high EGFR and GFAP expressions was detected due to destruction of nearby neurons (Soung and Klein 2019).

On the contrary, the histopathological analysis revealed that Sily could effectively neutralize the pathological disturbances associated with DA intake within the brain tissues and markedly minimize EGFR immunoreactivity, which is in agreement with the results that silibinin, a major constituent of Sily, could inhibit EGFR expression in human RCC cell lines (Liang et al. 2012). At the same time, blocking EGFR phosphorylation following Sily administration in the present study will be a good explanation for reducing GFAP expression (Li et al. 2014b).

Given the roles of TNF α and EGFR in amplifying neuroinflammatory responses through activating MAPKs, it is logical that animals that received DA either for 15 or 30 days would exhibit a considerable rise in the expression of MAPK entities, particularly ERK1/2, JNK, and p38. As a consequence, Sily supplementation could prohibit the phosphorylation of ERK1/2, JNK, and p38 MAPK, which is compatible with the data that Sily had a potent immunosuppressive effect on the activation of MAPKs cascade in microglia (Tian et al. 2019). Despite that MAPKs signaling pathway is essential for controlling neuronal cell survival, plasticity, and other biological functions (Qu et al. 2012), activating this pathway, especially in microglia, might trigger inflammatory responses and worsen neuronal degeneration (Eng and Ghirnikar 1994).

**Fig. 12:**
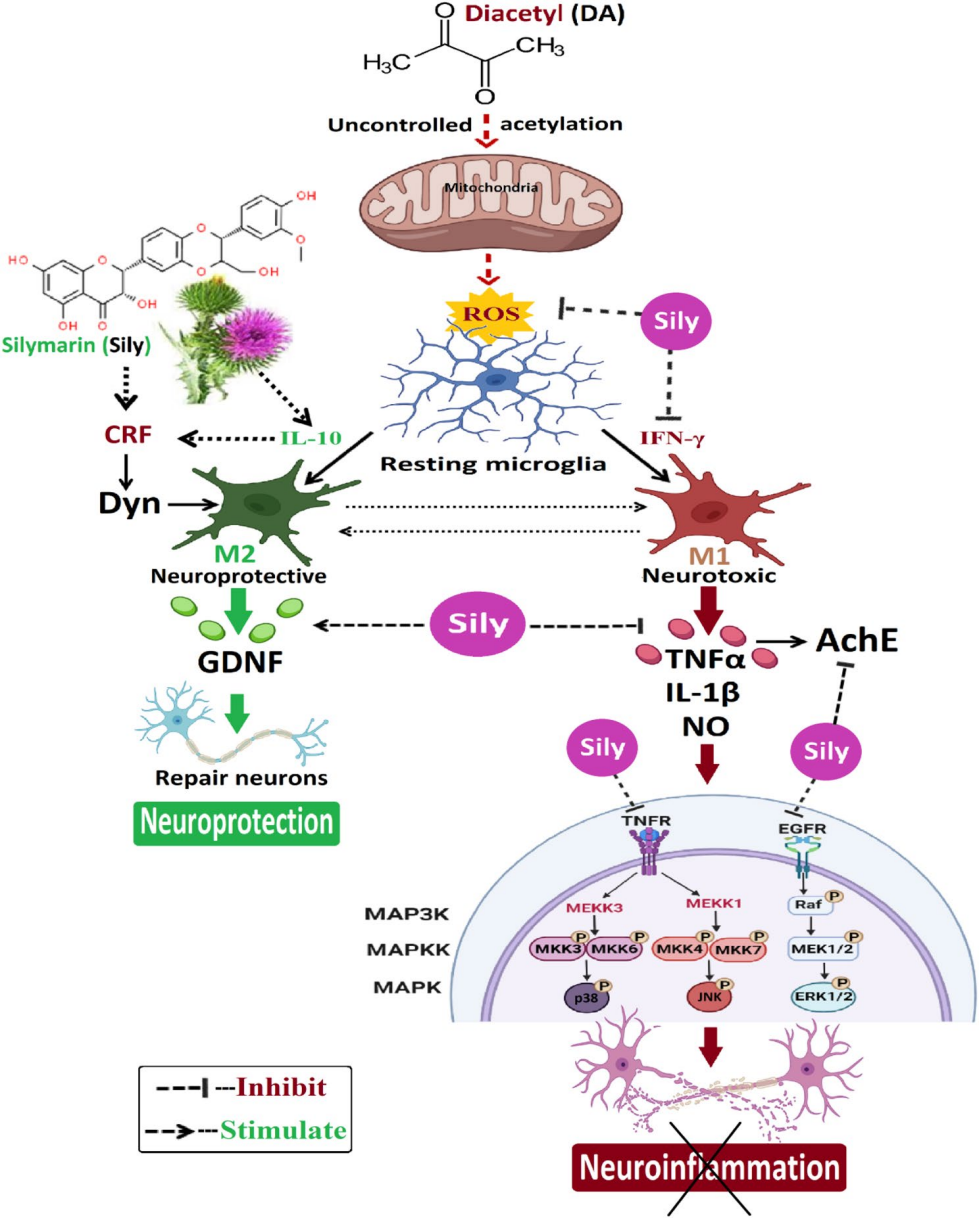
A schematic diagram showing the Sily-dependent protective and therapeutic strategies for DA-induced neuroinflammation. Following DA exposure, Sily may protect neurons against the DA-associated oxidative and inflammatory processes, as follows: (**i**) reducing ROS formation, (**ii**) boosting IL-10 secretion that initiates the M2 neuroprotective state either directly or indirectly by increasing CRF release, which then liberates Dyn and, as a consequence, promotes GDNF synthesis, (**iii**) blocking IFN-γ-mediated M1 neurotoxic state production and, as a result, inflammatory mediators release (TNF–α, IL-1β, and NO), as well as AChE activity, (**v**) depressing EGFR phosphorylation and, thereby, GFAP expression, and (**vi**) prohibiting the phosphorylation of ERK1/2, JNK, and p38 MAPK. *AChE* Acetylcholinesterase, CRF Corticotropin releasing factor**, ***Dyn* Dynorphin, *EGFR* Epidermal growth factor receptor, *GDNF* Glial cell line-derived neurotrophic factor, *GFAP *Glial fibrillary acidic protein, *IFN-γ* Interferon-γ, *IL-1β* Interleukin-1 beta, *IL-10* Interleukin-10, *MAPKs* Mitogen-activated protein kinases, *NO* Nitric oxide, *ROS* Reactive oxygen species, *TNFα* Tumor necrosis factor alpha

In conclusion, the results discussed the preventive and therapeutic efficacies of Sily as an influential agent revoking the oxidative–inflammatory cascade caused by DA in the brain. Because of its antioxidant and anti-inflammatory effects, Sily could preserve the cognitive and behavioral function, suppress the production of ROS, and stimulate IL-10-mediated M2 neuroprotective state either directly or indirectly by boosting CRF release and liberating Dyn. As a consequence, Sily promotes the secretion of neuroprotective markers such as GDNF as well as inhibits IFN-γ production, inflammatory mediator release such as TNF-α, IL-1β, and NO, and AChE activity. In addition, Sily could protect neurons from damage by down-regulating the expression of EGFR and GFAP by depressing ERK1/2, JNK, and p38 MAPK phosphorylation (Fig. 12), but only to a lesser extent when the animals were still given DA during therapy, indicating that early Sily treatment may reduce neuroinflammation development after DA consumption. These factors may explain, at least in part, the neuroprotective and neurotherapeutic roles that Sily may play against DA-induced neuroinflammation.

## Supplementary Information

Below is the link to the electronic supplementary material.

Supplementary file1 (MP4 982 kb)

Supplementary file2 (MP4 3854 kb)

Supplementary file3 (MP4 6900 kb)

Supplementary file4 (MP4 1464 kb)

Supplementary file5 (MP4 1293 kb)

Supplementary file6 (MP4 1974 kb)

Supplementary file7 (MP4 3479 kb)

## Data Availability

Inquiries about data availability should be directed to the authors.

## Abbreviations

AChE: Acetylcholinesterase
Dyn: Dynorphin
EGFR: Epidermal growth factor receptor
GDNF: Glial cell line-derived neurotrophic factor
GFAP: Glial fibrillary acidic protein
IFN-γ: Interferon gamma
IL-1β: Interleukin-1 beta
IL-10: Interleukin-10
MAPKs: Mitogen-activated protein kinases
MDA: Malondialdeyde
NO: Nitric oxide
ROS: Reactive oxygen species
TAC: Total antioxidant capacity
TNFα: Tumor necrosis factor alpha
